# A pulmonary tuberculosis outbreak in a long-term care facility

**DOI:** 10.1017/S0950268815002265

**Published:** 2015-11-23

**Authors:** C.-C. LAI, Y.-C. HSIEH, Y.-P. YEH, R.-W. JOU, J.-T. WANG, S.-L. PAN, H.-H. CHEN

**Affiliations:** 1 Emergency Department of Taipei City Hospital, Ren-Ai Branch, Taiwan; 2 Graduate Institute of Epidemiology and Preventive Medicine, College of Public Health, National Taiwan University, Taiwan; 3 Changhua Health Bureau, Taiwan; 4 Reference Laboratory of Mycobacteriology, Centre for Research, Diagnostic and Vaccine Development, Centres for Disease Control, Ministry of Health and Welfare, Taiwan; 5 Division of Infectious Diseases, Department of Internal Medicine, National Taiwan University Hospital, Taipei, Taiwan; 6 Department of Physical Medicine and Rehabilitation, National Taiwan University Hospital, Taipei, Taiwan

**Keywords:** Infectious period, latent period, LTBI, *Mycobacterium tuberculosis*, tuberculosis (TB)

## Abstract

In long-term care facilities (LTCFs), the elderly are apt to be infected because those with latent tuberculosis infections (LTBIs) are at an increased risk for reactivation and post-primary TB disease. We report an outbreak of TB in staff and residents in a LTCF. An outbreak investigation was conducted after two TB cases were reported from the LTCF. A tuberculin skin test (TST), bacteriological examination and chest radiograph were administered to all facility staff and residents. An outbreak is defined as at least two epidemiologically linked cases that have identical *Mycobacterium tuberculosis* genotype isolates. This outbreak infected eight residents and one staff member, who were confirmed to have TB in a LTCF between September 2011 and October 2012. Based on the Becker method, the latent and infectious periods were estimated at 223·6 and 55·9 days. Two initial TST-negative resident contacts were diagnosed as TB cases through comprehensive TB screening. Observing elderly people who have a negative TST after TB screening appears to be necessary, given the long latent period for controlling a TB outbreak in a LTCF. It is important to consider providing LTBI treatment for elderly contacts.

## INTRODUCTION

Although national implementation of the DOTS (directly observed treatment) strategy was launched in Taiwan in 2006, the incidence of tuberculosis (TB) remains high, at 54·5/100 000 person-years (p-yr) in 2011 [1]. The incidence rate of TB increases with age in Taiwan [2]. For people aged ⩾65 years, the incidence was almost 401·2/100 000 p-yr in 2011, which is nearly eight times the rate for the general population, and contributes more than half of the TB cases in Taiwan each year [1]. In ageing populations, TB remains a clinical and epidemiological challenge. In elderly patients, TB's clinical manifestation may present atypically, which may result in underdiagnosis of active cases [3]. In this population, TB outcomes after treatment are also unfavourable, as the success rate for elderly TB cases was only 60·3%, and the death rate was extremely high, reaching 32·1% in 2011 [1]. Because the elderly are prone to develop comorbid chronic diseases and disabilities [4], it has been estimated that about 10% of reported TB cases originate in long-term care facilities (LTCFs) [5].

Elderly individuals in LTCFs may be infected with TB due to primary infections or post-primary reactivation. However, molecular epidemiological studies found that secondary TB cases in the elderly were often attributed to TB sources in the same age groups, which suggests that there is the potential for recent or re-infection in the elderly, especially those who live in congregate settings [6]. Therefore, it is important to monitor TB's risk and spread in elderly people who are institutionalized to contain outbreaks of this disease. We describe an outbreak of TB in staff and residents in a LTCF in Changhua, Taiwan that occurred between September 2011 and October 2012.

## METHODS

Changhua County is located in the middle of Taiwan, with a population of ~ 1 300 000, of which 12·5% are aged ⩾65 years. Almost 0·7% of the Changhua population reside in LTCFs. In September 2011, a resident from a small LTCF was referred to a hospital and reported as a TB case (smear and culture positive). After diagnosing the index case, the local health authority performed a contact investigation according to the Taiwan Centres for Disease Control guidelines [7]. All the elderly residents are occasionally moved into different rooms to allow cleaning of their rooms. Such a measure would allow a positive case to have contact with all facility staff and residents. Close contact for such individuals is defined as sharing airspace with TB cases for >8 h/day or >40 h exposure. We therefore offered TB screening, including symptom review, chest radiography, sputum smear and culture for *Mycobacterium tuberculosis* and the tuberculin skin test (TST) for those meeting the criteria for close contact. All facility staff and residents were viewed as close contacts. The TST, which contained 2 tuberculin units of purified protein derivative (PPD) of the RT23 strain, was immediately performed and an induration ⩾10 mm indicated a positive result. The first TST was completed on 2 July 2012 and the second TST was completed on 18 September 2012.

Changhua's local health authority used genotyping for the early detection of the TB outbreak when ⩾2 cases of TB occurred within a 2-year period in the same congregate setting. *M. tuberculosis* isolates were sent to the National Reference Laboratory of Mycobacteriology for genotyping using IS*6110* restriction fragment length polymorphism (RFLP) [8, 9] and spacer oligonucleotide genotyping (spoligotyping) [10]. The RFLP and spoligotype were analysed using Bionumerics^®^ software, version 6.6 (Applied Maths, Belgium). Clustered cases, which were defined as isolates with matching strains, indicated recent transmission events [11]. When the first cluster of TB case occurs, there should be a contact investigation targeted to all facility staff and residents.

We defined an outbreak as at least two epidemiologically linked cases that are infected with identical genotypes of *M. tuberculosis* isolates. Definite TB cases were identified from clinical specimens, either by culture or molecular line probe assay. Furthermore, patients receiving a full course of TB treatment without bacteriological results were also defined as TB cases. Patients with latent tuberculosis infection (LTBI) are not compelled to receive treatment in Taiwan. However, we still provided LTBI treatment with the patients' consent.

We estimated the latent and infectious periods before symptom onset based on the exponential distributions of parameters *λ* and *β* with maximum-likelihood estimation (MLE), which assumed a fixed duration for the infectious period, as provided by the Becker method [12]. The likelihood function was obtained from this outbreak (see Supplementary material). The basic reproductive number (*R*_0_) was computed using the branching process [13, 14]. This study was approved by the Taipei City Hospital Institutional Review Board.

## RESULTS

**Fig. 1.**
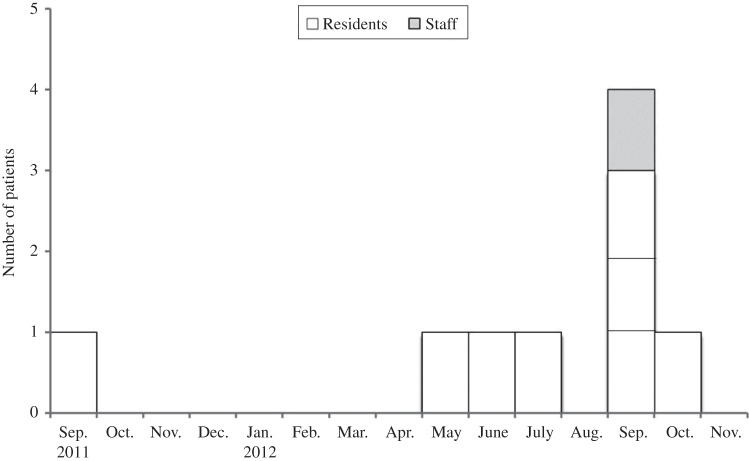
Epidemic data in chronological order for pulmonary tuberculosis cases in a long-term care facility.

Over a period of 13 months, nine TB cases (four cases smear-positive and culture-positive; three cases smear-negative and culture-positive; two cases smear-positive and culture-negative) were associated with a pulmonary TB outbreak between September 2011 and October 2012 (Fig. 1). Monitoring continued until September 2013. The pulmonary TB index case (smear-positive and culture-positive) without cavitation was diagnosed in September 2011. The contact investigation did not find any new TB cases. Eight months later, one cavitating TB case (smear-negative and culture-positive) and one active TB case (smear-positive and culture-positive) whose genotypes were identical to the index case were reported separately in May and June 2012. Therefore, the local health authority conducted a contact investigation in July 2012. In total, five TB cases were found by contact investigation and one TB case was reported during contact investigation.

**Fig. 2.**
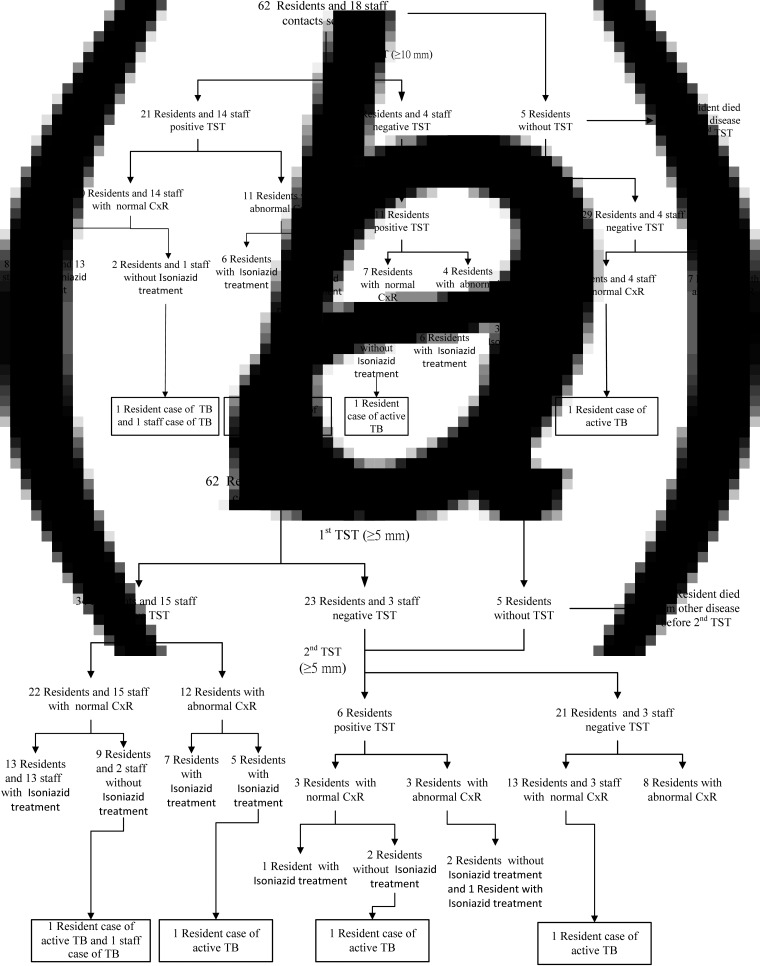
Flow chart for the contact investigation. (*a*) Positive tuberculin skin test (TST) results (⩾10 mm) were determined by CDC Taiwan guidelines. (*b*) Positive TST results (⩾5 mm) were determined by CDC US guidelines. One resident with the first negative TST died from a different illness before the second TST. Three confirmed cases and one suspected case were noted before or during the contact investigation period. Of the contacts with abnormal chest radiographs, only one case was associated with TB.

**Fig. 3.**
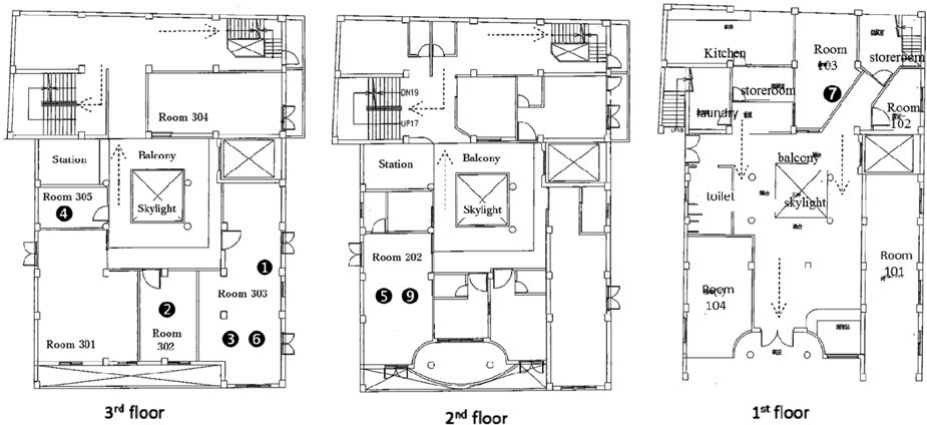
Room locations of residents with confirmed active cases during the investigation period in the long-term care facility. The index case was in room 303; the eighth case was a suspected staff member.

**Fig. 4.**
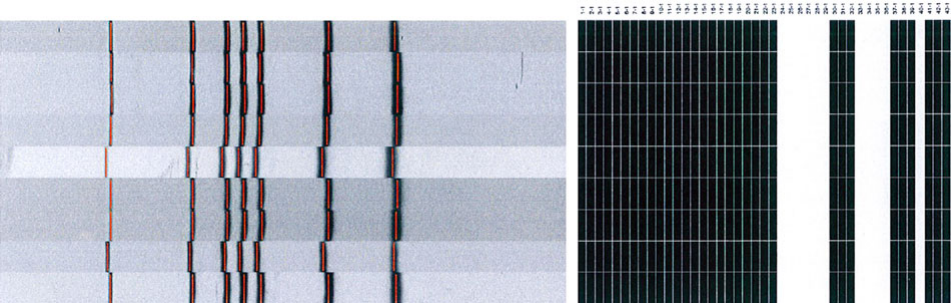
Patients with tuberculosis in the cluster had identical restriction fragment length polymorphism and spoligotyping patterns.

Although the LTCF is a three-storey building that houses 63 beds in 13 rooms, only 40 beds were registered and approved for use by the local health authority. The fresh air exchange rate was not high enough in this building, as there was no exhaust unit in the central air-conditioning system. There were 62 residents and 18 staff members in this LTCF during the investigation period, and the investigation results are shown in Figure 2*a*. One TST-negative resident contact was diagnosed as a TB case by the second TST, due to the alteration of the criterion for positive TST (⩾5 mm) in contacts by the US CDC guidelines (Fig. 2*b*) [15]. Of the contacts with abnormal chest radiographs, only one case was associated with TB. During the investigation period, four cases were located on the third floor of the LTCF and two cases were located on the second floor (Fig. 3). By tracing the cases' histories, we found that these cases and the index case had contact with each other in the same room on the third floor. In addition, the resident case that was located on the first floor had been cared for by the staff member who was a case and who had contact with the third-floor cases. Resident and staff characteristics are listed in Table 1. The new TST conversion rate was 25·0% in residents and 0% in staff. All resident cases, except for the first-floor case, had been in contact with each other in the same room during the communicability period. Of the TB cases, the first five resident cases presented with a fever, while the other cases had no symptoms. Sputum smears were positive for TB, but the sputum culture had no growth in one resident and one staff case. In addition, the RFLP and spoligotyping results showed that the active cases were infected with identical genotypes of *M. tuberculosis* (Fig. 4).

**Table 1.** Tuberculosis contact investigation in a long-term care facility

	Residents (*N* = 62)	Staff (*N* = 18)
Age, yr, mean (s.d.)	75·9 (11·8)	35·5 (12·2)
Gender, %		
Male	21 (33·9)	2 (11·1)
Female	41 (66·1)	16 (88·9)
Positive TST (first^)^*, *n*/*N* (%)	21/57 (36·8)	14/18 (77·8)
Positive TST (second)† *n*/*N* (%)	11/41 (26·8)	0/4 (0·0)
New TST conversion, *n*/*N* (%)	9/36 (25·0)	0/4 (0·0)
Tuberculosis cases, *n* (%)	4 (6·5)	1 (5·6)

TST, Tuberculin skin test.

* The first TST was done on 2 July 2012.

† The second TST was done on 18 September 2012.

Positive criteria of TST is determined by ⩾10 mm.

The incidence rate was 6060·6/100 000 p-yr for residents and 2777·8/100 000 p-yr for staff. The resident cases were bedridden due to stroke (*n* = 5, 62·5%), chronic obstructive pulmonary disease (COPD) with hypoxic encephalopathy (*n* = 3, 37·5%) and bladder cancer with distal metastasis (*n* = 1, 12·5%). In addition, half of the residents required tracheostomy suction, while the other half required oral and nasal tracheal suction. The TB strain was susceptible to all first-line anti-TB drugs. However, five resident cases died during treatment. Of the total 46 LTBI cases, only 26 agreed to receive treatment. Two cases died from other causes during treatment and one case stopped treatment due to side-effects.

The latent period was about 223·6 days [the estimated parameter of latent period (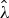) = 0·0045 (2·17 × 10^−6^), see Supplementary material] and the infectious period before symptom onset was about 55·9 days [the estimated parameter of infectious period before symptom onset (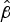) = 0·0179 (3·47 × 10^−5^), see Supplementary material]. Hence, the incubation period was about 279·5 days. According to our latent period estimation, there were at least two generations and three generations at most. In this cluster, *R*_0_ ranged between 0·9739 and 0·9796.

Control measures, including contact tracing and case follow-ups, were performed by the facility with the local public health authority's assistance. These measures included closing the facility to admissions, increasing ventilation rates in the building and decreasing the numbers of residents per room.

As a matter of course, all suspected or confirmed TB disease cases should be placed in an isolated room or transferred to the hospital for treatment. All contacted residents and staff members received chest radiograph examinations every 6 months for 2 years after the last confirmed TB case. Sputum cultures should be conducted when contacts have suspected TB symptoms.

## DISCUSSION

The outbreak of TB in a LTCF is unsurprising, albeit rarely reported, particularly if there is a lack of active intervention and treatment given the presence of high-risk factors (e.g. cavitary disease; suctioning and aerosolization; confined, crowded and poorly ventilated facility; elderly contacts with severe pulmonary comorbidities). In the current LTCF reported here, there were nine confirmed cases associated with a pulmonary TB outbreak. The TST contact positive rate was 51·6% (32/62) and 77·8% (14/18) in residents and staff. By comparison, TB contacts had a 69% positive TST response in a community-based study in Taiwan [16]. However, the new TST conversion rate was 25·0% for residents. LTBI treatment should be administered in bacille Calmette-Guérin (BCG)-vaccinated populations, which has been suggested in previous studies [17, 18].

The Reference Laboratory at Taiwan CDC has adopted three genotyping methods, spoligotyping, 15-loci MIRU-VNTR (mycobacterial interspersed repetitive units–variable number tandem repeats), and RFLP for assisting TB outbreak investigations. Beijing, Haarlem and East African-Indian (EAI) were the three major spoligotypes observed in Taiwan [19, 20]. It was found that even 24-loci MIRU-VNTR was not feasible for typing Beijing family strains compared to the RFLP method [21, 22]. Therefore, RFLP genotyping remains the gold standard with the highest discriminatory power. The genotype identified in this episode was not a common spoligotype circulating in Taiwan. Genotyping may help to identify small clusters that could become outbreaks and define areas for location-based TB screenings [23–26]. Hence, use of genotyping can enhance TB outbreak monitoring and target interventions (e.g. intensified contact investigations). However, previous studies that used genotype monitoring did not indicate that LTCFs were at a high risk for TB outbreaks. This strategy allowed us to identify which LTCFs were high-risk targets.

There is a trade-off between the risk of transmission and the scale of screening. We noted two sputum culture-positive TB cases with normal chest radiographs in this outbreak, which was not uncommon [27]. Sputum cultures should be performed in symptomatic TST-positive contacts, with cough and fever, for early detection [27, 28]. However, the symptoms of TB in the elderly are heterogeneous, ranging from the absence of any symptoms to a fulminant course [29]. Whether a certain symptom is more or less frequent in the elderly is hard to determine [30, 31]. In addition, the older residents of the LTCF susceptible to active TB might not present the typical symptoms of cough or fever because of a worsened general health status with multiple comorbidity and severe disability, such as stroke and COPD, most of which require tracheostomy suction or oral and nasal tracheal suction. In addition, it is important to diagnose active TB early in individuals who initially have negative TST results in order to reduce transmission in LTCFs. One TST-negative resident contact was diagnosed as a TB case in the second TST, even when the positive criterion of TST was altered to ⩾5 mm. Hence, when the first clustered case defined by genotyping was found, the ongoing recent transmission of TB might have already occurred. This strongly necessitates screening by sputum culture and chest radiograph despite negative TST results in LTCFs. Furthermore, it is important to provide LTBI treatment for elderly contacts who have comorbidities regardless of LTBI test results. The above findings were due to lower sensitivity to TST in the elderly [32–36]. Testing by interferon-gamma release assay (IGRA) may be considered in the elderly. However, we were concerned about adverse medication reaction risks for LTBIs in the elderly population. Therefore, further research should examine IGRA testing and LTBI treatment tolerance in elderly populations that have high comorbidity in LTCFs or other healthcare settings.

Studies that estimate TB's latent and infectious periods are rare. In this outbreak, the infectious period before symptom onset was about 2 months. Experts recommend that the infectious period be defined as 3 months prior to symptom onset [15]. The latent period was about 32 weeks. Contact investigations and LTBI treatments become important strategies to control and eliminate TB during the latent period. Our study had shorter incubation periods compared to previous studies (45% within 1 year; median 1·26 years) [37], which may be partially attributed to the outbreak and partially due to bias resulting from the 2-year follow-up period.

When *R*_0_ > 1, the number of persons with TB increases, and when *R*_0_ < 1, that number declines. The reported estimate of *R*_0_ in the current study is not a basic reproductive number, i.e. the natural course of the spread of infection in the absence of intervention, but something analogous to an effective *R*_0_ (often used in vaccination), as a result of the provision of infection control such as active surveillance, isolation, active treatment, and improvement of air quality) immediately after the outbreak and investigation. There is no available basic reproductive number estimate of TB in LTCFs for comparison in Taiwan. Only one study reported that the median value of overall *R*_0_ in eastern Taiwan ranged from 1·65 to 1·72 [38] without active intervention in the community-based setting rather than LTCF. As that study's estimate also included reactivation and slow TB progression (longer than 2 years) the comparison of *R*_0_ in different settings should be made with great caution. Our reported effective reproductive number estimate that was <1 implies that the outbreak of TB infection died out in the LTCF because infection controls (including active surveillance, isolation, treatment, and improvement of the air quality) were performed.

There are several limitations to this study. Without two-step testing to measure tuberculin reactivity and no documented baseline TST results within the previous 12 months, it is possible to have false-negative TST results because boosting is more common in the elderly and occurs in 15% of screened elderly subjects [39]. False-positive TST results are uncommon due to an atypical mycobacteria infection or following BCG vaccination. The effects of the BCG remote vaccination for a positive tuberculin response in adults aged >30 years is negligible [16]. LTBI or *M. tuberculosis* infections should be assumed in older patients who have positive TST or positive IGRA results. However, IGRAs were not measured in this outbreak. In addition, the air exchange rate was low in the field investigation, but we did not measure the number of air changes per hour.

Aerosolization of the TB patient's secretions from repeated suctioning and inadequate room ventilation were routes for the spread of TB in this outbreak. Prior investigations of TB outbreaks in healthcare settings have found similar transmission routes [28]. We inferred that sputum suction and low air ventilation rates were risk factors for TB transmission in residents in this outbreak.

In conclusion, this outbreak reinforced the importance of comprehensive TB screening, including sputum examinations and chest radiographs, for all residents and staff regardless of negative TST results when the first clustered case, as defined by genotyping, was found or when the symptoms of the elderly residents were hard to clarify due to the complicated disease/disability status. LTBI treatment for elderly contacts who have positive TST or positive IGRA results should be considered if there is acceptable treatment tolerance in elderly populations with high comorbidity.

## Supplementary material

For supplementary material accompanying this paper visit https://doi.org/10.1017/S0950268815002265. click here to view supplementary material
