# Supplementary material for: A pulmonary tuberculosis outbreak in a long-term care facility
Source: Epidemiol Infect. 2015 Nov 23;144(7):1455–62. doi: 10.1017/S0950268815002265 (PMC4823835; doi:10.1017/S0950268815002265)
Supplement: Supplementary file 1 [file S0950268815002265sup001.doc]

**Supplementary Material**

Let variable U denote the time to infectious period and variable W denote the time to the onset of symptoms. So Y=W-U denote the duration of infectious period before symptoms onset. Let X denote the duration of latent period. In this outbreak, one introductory case and 4 secondary cases with symptoms were found. We assumed X and Y both follow the exponential distribution and the parameters were λ and β, respectively. We also assumed that the duration of infectious period (μ) is fixed before the onset of symptoms. If the duration of latent period has density function,$f_{x}(x)$. Therefore, we have the likelihood function:

$L(\beta,\lambda|w)=\prod_{i=2}^{5} \int_{0}^{\mu} \beta e^{-\beta x}f_{x}(w_{i}-w_{1}-x)dx$, then

$$\begin{matrix} \int_{0}^{\mu} \beta e^{-\beta x}\lambda^{4}e^{-\lambda[(220-x)+(260-x)+(306-x)+(332-x)]}dx \\ =\beta\lambda^{4}\int_{0}^{\mu} e^{-1118\lambda+(4\lambda-\beta)x}dx \end{matrix}$$

1. Given $4\lambda-\beta=0$,

Therefore,

E(X)=1118/5=223.6 days, E(Y)=1118/20=55.9 days

and E(X+Y)=279.5 days.

$$I(\lambda)=\frac{d^{2}L}{d\lambda^{2}}=80\lambda^{3}\mu e^{-1118\lambda}-24\text{*}1118\lambda^{4}\mu e^{-1118\lambda}+4\text{*}1118^{2}\lambda^{4}\mu e^{-1118\lambda}$$

We can get the variance of λ and β from inverse expected information matrix:

$\text{Var}\left(\lambda\right)=2.41*10^{-8}\hat{\mu}=2.17*10^{-6}\begin{matrix} & (if\hat{\mu}=90) \end{matrix}$. Similarly, we have,

$$\text{Var}\left(\beta\right)=Var(4\lambda)=16*\begin{matrix} 2.17*10^{-6}=3.47*10^{-5} & \end{matrix}$$

1. If $4\lambda-\beta\neq0$

$$\frac{dL}{d\beta}=4\hat{\beta}\hat{\lambda}^{3}(3\hat{\lambda}-\hat{\beta})e^{(4\hat{\lambda}-\hat{\beta})\hat{\mu}}-4\hat{\beta}\hat{\lambda}^{3}(3\hat{\lambda}-\hat{\beta})+[\hat{\beta}\hat{\lambda}^{4}(4\hat{\lambda}-\hat{\beta})(4\hat{\mu}-1118)]e^{(4\hat{\lambda}-\hat{\beta})\hat{\mu}-1118\hat{\lambda}}$$

$$\frac{dL}{d\lambda}=\hat{\lambda}^{4}(1+\hat{\beta}+4\hat{\lambda}\hat{\beta}-\hat{\beta}^{2})[e^{4(\hat{\mu}-1118)\hat{\lambda}-\hat{\beta}\hat{\mu}}-1]$$

Let $\frac{dL}{d\beta}=\frac{dL}{d\lambda}=0$, we can get

(a) If$\hat{\lambda}=0,\hat{\beta}\text{is arbitrary.}$

(b) If $1+\hat{\beta}+4\hat{\lambda}\hat{\beta}-\hat{\beta}^{2}=0$, then $\hat{\beta}=\frac{1+\sqrt{5}}{2}=1.62,\begin{matrix} & \end{matrix}\hat{\lambda}=0$;

(c) if $4(\mu-1118)\lambda-\beta\mu=1$, then it is not admissible.

All parameters (β,μ,λ) must be positive. Therefore, we got the solution, $\hat{\lambda}=\frac{5}{1118},\begin{matrix} & \end{matrix}\hat{\beta}=\frac{20}{1118}$.
